# Risk Evaluation Model of Highway Tunnel Portal Construction Based on BP Fuzzy Neural Network

**DOI:** 10.1155/2018/8547313

**Published:** 2018-08-28

**Authors:** Xianghui Deng, Tian Xu, Rui Wang

**Affiliations:** School of Civil and Architecture Engineering, Xi'an Technological University, Xi'an 710032, China

## Abstract

Risk assessment for tunnel portals in the construction stage has been widely recognized as one of the most critical phases in tunnel construction as it easily causes accident than the overall length of a tunnel. However, the risk in tunnel portal construction is complicated and uncertain which has made such a neural network very attractive to the construction projects. This paper presents a risk evaluation model, which is obtained from historical data of 50 tunnels, by combining the fuzzy method and BP neural network. The proposed model is used for the risk assessment of the Tiefodian tunnel. The results show that the risk evaluation level is IV, slope instability is the greatest impact index among four risk events, and the major risk factors are confirmed. According to the evaluation results, corresponding risk control measures are suggested and implemented. Finally, numerical simulation is carried out before and after the implementation of risk measures, respectively. The rationality of the proposed risk evaluation model is proved by comparing the numerical simulation results.

## 1. Introduction

Over the past few decades, construction of highways has been developing quickly in China. Tunnel construction has become the first choice for highway alignment because of its advantages of optimal alignment, reduced travelling time, and enhanced operation efficiency. Meanwhile, tunneling is a dangerous occupation owing to its complicated construction technology, uncertainties risk factors, and complicated geological conditions. There are numerous casualties and millions of economic losses caused by tunnel accidents. Compared with the overall length of a tunnel, portals usually have a limited area of influence. The weathering extent of surrounding rock is heavier, the buried depth is shallow, and it is vulnerable to the impact of rainfall. In the entrance section, the construction is difficult, and these unfavorable factors easily lead to engineering accidents, such as slope instability, large deformation, tunnel collapse, and other accidents [1]. In most instances, risk assessment may reduce the probability of accidents and decrease economic costs. Therefore, it is necessary to take the modern risk management method to evaluate the risk of the tunnel portal construction process.

Risk assessment for highway tunnel portals during the construction stage has been widely recognized as one of the most critical phases. However, risk factors of tunnel portal construction are complex and uncertain. At the same time, most of the information in risk evaluation comes from the expert's subjective judgement which is usually imprecise and subjective in the decision-making process. Handling vagueness and subjectivity becomes a primary task in risk assessment.

The fuzzy system describes a class of extrapolated blurring without explicit boundaries and establishes a correspondence between uncertainties and membership functions so that favorable mathematical tools can be used to analyze many inaccurate vague phenomena in nature. Fuzzy theory has found in-depth research and application in the mining, nuclear, petrochemical, and construction industries. van Laarhoven and Pedrycz introduced the concept of fuzzy set theory into the traditional analytic hierarchy process (AHP) and originally proposed the fuzzy analytic hierarchy process (FAHP) [2, 3]. Fuzzy hierarchy evaluation is a synthetic risk evaluation method based on AHP and fuzzy comprehensive evaluation [4, 5]. It is well known as a useful tool to deal with imprecise, uncertain, or ambiguous data and the high nonlinearity and complexity [6–8]. The uncertain comparison judgement can be represented by the fuzzy number. To deal with the uncertainty or vagueness of data, the fuzzy analytic hierarchy process (FAHP) has found huge applications in recent years [9]. Compared with the overall length of a tunnel, portals usually have the complicated terrain and poor geological conditions. Thus, the construction of a tunnel portal is often difficult and easily leads to engineering accidents [10]. Wang et al. applied the logarithmic fuzzy preference programming (LFPP) method to analyze the data [11]. Although FAHP has good applicability in construction industries than traditional risk assessment methods such as AHP, it still has subjectivities to identify the weight and set a single-factor judgement matrix in risk evaluation.

Artificial neural network (ANN) is also known as a neutral network which is a mathematical model for finding patterns among datasets where there are complex relationships between the inputs and outputs. ANN attempts to simulate the structure and operation of the human neural network system. Since ANN acts like a “black-box” and cannot explain the reasoning process, it can well achieve the self-adaptation through the learning function and can acquire the fuzzy data expression knowledge accurately and automatically. The ability to learn from examples has made this technique a very useful tool in data modeling [12–14]. In the past few decades, researchers applied ANN in many aspects of construction management such as risk analysis, resource optimization, and productivity assessment [15]. ANN is capable of learning from the data; however, it cannot explain the quality of the input-output mapping process. On the contrary, the fuzzy system is a systematic reasoning method that is more compatible with human logic and intuition.

ANN and fuzzy theory are complementary technologies. Fuzzy theory tries to describe and deal with the ambiguity concept in human language and thinking. The artificial neural network is based on the human brain's physiological structure and information-processing process. With the rapid development of fuzzy system and artificial neural network research, it has been found that the original independent field can be compensated and fused together, which leads to a new field—fuzzy neural network (FNN). FNN provides effective tools for addressing uncertainties in decision-making [16]. In the past few years, this system has been widely applied to develop risk management models in the engineering construction. Wang et al. combined AHP with the backpropagation (BP) neural network (which is a multilayer error-feedforward network), and they proposed a model of coal mine water disaster emergency logistics risk assessment [17]. Mirahadi and Zayed developed a modified neural-network-driven fuzzy reasoning (NNDFR) model with optimized parameters and improved the developed model so that it can simultaneously deal with crisp values and fuzzy numbers [18]. Li et al. proposed an analytic hierarchy process model for the transformer risk assessment built by a transformer risk assessment method based on FAHP and artificial neural network (ANN) [19].

For the purpose of handling the vagueness and subjectivity in risk evaluation of highway tunnel portal construction, this paper proposes a risk evaluation model by combining fuzzy theory with the neural network. There are many kinds of neural network types. This paper uses the BP network—a multilayer feedforward neural network—which can achieve any nonlinear mapping from the input to output. 80% to 90% of the neural network model uses the BP network or its change form. The feasibility and effectiveness of this model are proved by an engineering case.

The remainder of this research is organized as follows: Section 2 introduces the BP fuzzy neutral network model in detail.  Section 3 shows the case study.  Section 4 verifies the accuracy of the model. This paper is concluded in the last section.

## 2. BP Fuzzy Neutral Network Model Development

### 2.1. Design of the Topology Structure

**Figure 1:**
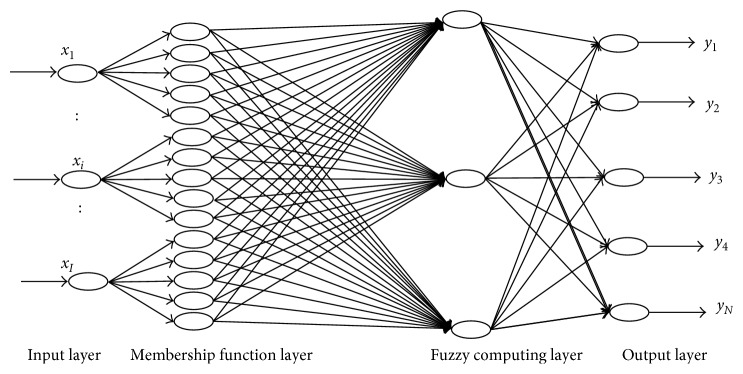
Structure of the BP neural network (*x*_*I*_: input variable; *y*_*N*_: estimated output).

The BP neural network generally has three or more layers of neurons. There are input layer, hidden layer, and output layer, respectively. According to the Kolmogorov theorem, this model uses a three-layer BP neural network with a single hidden layer [20]. There are some debates about the relevance of Kolmogorov's theorem. Girosi and Poggio have criticized this interpretation of Kolmogorov's theorem. They reviewed Kolmogorov's theorem on the representation of functions of several variables in terms of functions of one variable and showed that it is irrelevant in the context of networks for learning. However, Kůrková supported the relevance of this theorem to neural nets. He gave a direct proof of the universal approximation capabilities of perceptron-type networks with two hidden layers by taking advantage of techniques developed by Kolmogorov. He proved the feasibility of the Kolmogorov theorem in the BP neural network [21, 22]. The topology was built with the BP neural network combined with the characteristics of the highway tunnel portal construction, which is shown in Figure 1.

This topology is divided into four parts: the first part is the input layer and each of its nodes represents an input variable. The second part is the fuzzy process. The neural network input value, which is the input layer of the neural network structure, can be obtained by the fuzzification of the risk factor. The third part is the fuzzy reasoning layer, which is the hidden layer. It can complete the mapping between the input variable and the fuzzy value of the output variable. The fourth part is the output layer, which is the result of the risk level.

### 2.2. Hierarchical of the Highway Tunnel Portal Construction Risk

The evaluation of the index system is the basis and key of the risk assessment research. It directly affects the objectives, accuracy, and results of the evaluation. From risk evaluation theory of the highway tunnel construction, any risk factors associated with a highway tunnel project are likely to evolve into a risk event, leading to the occurrence of construction safety accidents. This paper collected the data of the 50 highway tunnel samples from the literature.

**Figure 2:**
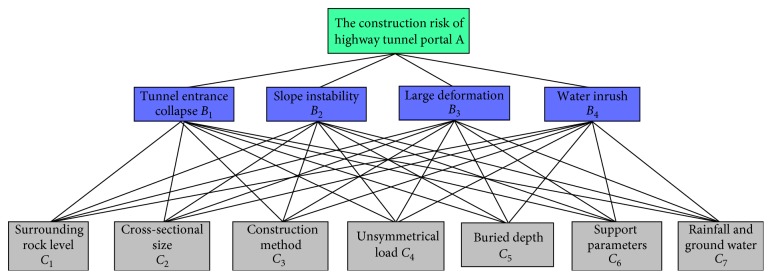
The hierarchical structure of risk evaluation in highway tunnel portal construction.

All these 50 tunnels are located in China. Among them, 12 tunnels are located in Fujian Province, 10 in Shanxi Province, 8 in Zhejiang Province, 6 in Shaanxi Province, 5 in Hebei Province, 3 in Shandong Province, 3 in Hubei Province, and 3 in Hunan Province. There are 39 two-lane and 11 three-lane tunnels among 50 tunnels. There are 15 tunnels with a length of 3000 m or more, 22 tunnels with a length of 1000–3000 m, and 13 tunnels with a length of less than 1000 m. The longest one is the Mayazi tunnel which is 9007 m. The Wulidun tunnel is the shortest one which is about 980 m. According to the safety status and the screening of risk factors, the risk assessment index system of the 50 highway tunnel portal construction was built, as shown in Figure 2.

It is composed of three layers. On the top, the goal of this paper is to assess the risk level of highway tunnel portal construction. The first index is four risk events of the highway tunnel portal construction, which include tunnel entrance collapse (*B*_1_), slope instability (*B*_2_), large deformation (*B*_3_), and water inrush (*B*_4_). The second index is seven risk factors, which are surrounding rock level (*C*_1_), cross-sectional size (*C*_2_), construction method (*C*_3_), unsymmetrical load (*C*_4_), buried depth (*C*_5_), support parameters (*C*_6_), and rainfall and groundwater (*C*_7_).

### 2.3. Determination of the Sample Data of the Evaluation Model with the Fuzzy Method

#### 2.3.1. Modeling Input

It is necessary to quantify these risk indexes of the established index system because the BP neural network requires quantitative data in risk evaluation of construction. As a result, the fuzzy evaluation method was used to quantify the degree of risks in this research. Take one of the 50 tunnels as an example.

  Step 1: the set of the comments level

In this paper, the risk of highway tunnel portal construction was divided into five levels according to 50 sample tunnels, where level I, level II, level III, level IV, and level V represent less risk, low risk, general risk, high risk, and higher risk, respectively. The reviews set is expressed by *V* as follows:

(1) $$\begin{array}{c} V=\left\{\text{I},\;\;\mathrm{II},\;\;\mathrm{III},\;\;\mathrm{IV},\;\;V\right\}. \end{array}$$

  Step 2: factors domain of the evaluation object

The number of evaluation objects is *n*. Each first index corresponds to seven evaluation results. The factor set is expressed by *U* as follows:

(2) $$\begin{array}{c} U=\left\{u_{1},\;\;u_{2},\;\;\ldots ,\;\;u_{n}\right\}. \end{array}$$

  Step 3: membership matrix

Take Tunnel 1 which belongs to the 50 tunnels as an example.

Each risk factor holds a degree of membership to every risk level for different risk events, with the summation of all of its degrees of membership being 1. The membership matrices of the secondary indicators at each level are as follows:

(3) $$\begin{array}{c} R_{1}=\left[\begin{array}{ccccc} 0 & 0.2 & 0 & 0.6 & 0.2\\ 0 & 0 & 1 & 0 & 0\\ 0.8 & 0.2 & 0 & 0 & 0\\ 1 & 0 & 0 & 0 & 0\\ 0.6 & 0.4 & 0 & 0 & 0\\ 0 & 0.7 & 0.3 & 0 & 0\\ 0.8 & 0.2 & 0 & 0 & 0 \end{array}\right],\\ R_{2}=\left[\begin{array}{ccccc} 1 & 0 & 0 & 0 & 0\\ 0 & 0.7 & 0.3 & 0 & 0\\ 0 & 1 & 0 & 0 & 0\\ 0.9 & 0.1 & 0 & 0 & 0\\ 1 & 0 & 0 & 0 & 0\\ 0 & 0.4 & 0.6 & 0 & 0\\ 1 & 0 & 0 & 0 & 0 \end{array}\right],\\ R_{3}=\left[\begin{array}{ccccc} 0 & 0 & 0.6 & 0.4 & 0\\ 0 & 0.3 & 0.7 & 0 & 0\\ 0.7 & 0.3 & 0 & 0 & 0\\ 0 & 1 & 0 & 0 & 0\\ 0 & 1 & 0 & 0 & 0\\ 0 & 0.1 & 0.9 & 0 & 0\\ 0.6 & 0.4 & 0 & 0 & 0 \end{array}\right],\\ R_{4}=\left[\begin{array}{ccccc} 1 & 0 & 0 & 0 & 0\\ 1 & 0 & 0 & 0 & 0\\ 1 & 0 & 0 & 0 & 0\\ 0.6 & 0.4 & 0 & 0 & 0\\ 0.2 & 0.8 & 0 & 0 & 0\\ 0.3 & 0.7 & 0 & 0 & 0\\ 0.1 & 0.9 & 0 & 0 & 0 \end{array}\right], \end{array}$$

where **R**_1_ is the degree of membership of each second index to tunnel entrance collapse, **R**_2_ is the degree of membership of each second index to slope instability, **R**_3_ is the degree of membership of each second index to large deformation, and **R**_4_ is the degree of membership of each second index to water inrush. Each row in **R** represents the membership degree of each index to the risk probability levels I, II, III, IV, and V. The above four membership matrices are combined to obtain a comprehensive evaluation factor of the membership matrix **R**. This membership matrix is represented as follows:

(4) $$\begin{array}{c} R=\left[\begin{array}{c} R_{1}\\ R_{2}\\ R_{3}\\ R_{4} \end{array}\right]. \end{array}$$

  Step 4: the weight of the risk level

Each risk level has different impacts on the highway tunnel portal construction; therefore, the weight of each risk level in the judging set is different in the set of the comments level. Determine the standard risk probability level *V*={I, II, III, IV, V} = {very unlikely, impossible, accidental, possible, most likely} with a weight of {1/25, 3/25, 5/25, 7/25, 9/25} which is as follows:

(5) $$\begin{array}{c} W=\left(w_{\text{I}},\;\;w_{\text{II}},\;\;w_{\text{III}},\;\;w_{\text{IV}},\;\;w_{\text{V}}\right)=\left(\frac{1}{25},\;\;\frac{3}{25},\;\;\frac{5}{25},\;\;\frac{7}{25},\;\;\frac{9}{25}\right), \end{array}$$

where (*w*_I_, *w*_II_, *w*_III_, *w*_IV_, *w*_V_) means the weight of the risk level from I to V.

  Step 5: calculate modeling input

The input data of the risk evaluation model can be obtained by combining **R** and **W**, which are as follows:

(6) $$\begin{array}{c} B_{X}=W\cdot R=\left(\begin{array}{c} 0.136,\;\;0.200,\;\;0.344,\;\;0.360,\;\;0.328,\;\;0.256,\;\;0.344,\;\;0.360,\;\;0.256,\;\;0.280,\;\;0.352,\;\;0.360,\;\;0.232,\;\;0.360\\ 0.168,\;\;0.224,\;\;0.336,\;\;0.280,\;\;0.280,\;\;0.208,\;\;0.328,\;\;0.360,\;\;0.360,\;\;0.360,\;\;0.328,\;\;0.296,\;\;0.304,\;\;0.288 \end{array}\right), \end{array}$$

where **B**_*X*_ is the weight of seven second indexes corresponding to four different first indexes, which indicates the impact of risk factors. The value of **B**_*X*_ is in the range (0, 1), which can be used as the input of the neural network.

The input data of Tunnel 1 are calculated as shown in Table 1.

Similar to the abovementioned calculation process, the input data of the other 49 highway tunnels can be obtained. The calculation process is omitted here due to the space constraints.

#### 2.3.2. Modeling Outputs

The expected output corresponding to this paper is the risk evaluation level of highway tunnel portal construction. In this section, FAHP is used to calculate the risk evaluation level of Tunnel 1 portal construction. The calculation process of the output data for Tunnel 1 will be described in detail as follows.

  Step 1: calculate the weight

AHP is employed to calculate the weight of the risk index of highway tunnel portal construction. According to the abovementioned hierarchical structure, individual judgements are collected and comparison matrices of the risk factor are constructed. Calculation of the comparison matrix requires consistency checking. The expression's consistency ratio (CR) can be computed as follows:

(7) $$\begin{array}{c} \text{CR}=\frac{\left(\lambda_{\text{m}}-n\right)/\left(n-1\right)}{\text{RI}}, \end{array}$$

where *λ*_m_ is the maximum eigenvalue of the judgement matrix, *n* is the matrix order, and RI is the average random consistency indicator which is shown in Table 2. If CR < 0.1, the comparison matrix is acceptable. Otherwise, experts' judgements should be adjusted until CR < 0.1.

Through expert surveys and associated risk theories, a judgement matrix for the construction stage of the cavern section was established for each of the identified seven risk factors. The comparison matrix **A** and CR of the risk event are as follows:

(8) $$\begin{array}{c} A_{B}=\left[\begin{array}{cccc} 1 & 3 & 7 & 9\\ \frac{1}{3} & 1 & 3 & 7\\ \frac{1}{7} & \frac{1}{3} & 1 & 3\\ \frac{1}{9} & \frac{1}{7} & \frac{1}{3} & 1 \end{array}\right],\\ \text{CR}=0.0363<0.1, \end{array}$$

where **A**_*B*_ is the comparison matrix of the A-B-level risk. The comparison matrices **B** and CR of risk factors are as follows:

(9) $$\begin{array}{c} B_{1}=\left[\begin{array}{ccccccc} 1 & 3 & \frac{1}{3} & 11 & 9 & 7 & 5\\ \frac{1}{3} & 1 & \frac{1}{5} & 9 & 7 & 5 & 3\\ 3 & 5 & 1 & 13 & 11 & 9 & 7\\ \frac{1}{11} & \frac{1}{9} & \frac{1}{13} & 1 & \frac{1}{3} & \frac{1}{5} & \frac{1}{7}\\ \frac{1}{9} & \frac{1}{7} & \frac{1}{11} & 3 & 1 & \frac{1}{3} & \frac{1}{5}\\ \frac{1}{7} & \frac{1}{5} & \frac{1}{9} & 5 & 3 & 1 & \frac{1}{3}\\ \frac{1}{5} & \frac{1}{3} & \frac{1}{7} & 7 & 5 & 3 & 1 \end{array}\right],\\ {\text{CR}}_{1}=0.0766<0.1,\\ B_{2}=\left[\begin{array}{ccccccc} 1 & 10 & 6 & 8 & 4 & \frac{1}{16} & \frac{1}{4}\\ \frac{1}{10} & 1 & \frac{1}{5} & \frac{1}{3} & \frac{1}{7} & \frac{1}{13} & \frac{1}{11}\\ \frac{1}{6} & 5 & 1 & 3 & \frac{1}{3} & \frac{1}{9} & \frac{1}{7}\\ \frac{1}{8} & 3 & \frac{1}{3} & 1 & \frac{1}{5} & \frac{1}{11} & \frac{1}{9}\\ \frac{1}{4} & 7 & 3 & 5 & 1 & \frac{1}{7} & \frac{1}{5}\\ 6 & 13 & 9 & 11 & 7 & 1 & 3\\ 4 & 11 & 7 & 9 & 5 & \frac{1}{3} & 1 \end{array}\right],\\ {\text{CR}}_{2}=0.0995<0.1,\\ B_{3}=\left[\begin{array}{ccccccc} 1 & 3 & 11 & 13 & 7 & 5 & 9\\ \frac{1}{3} & 1 & 10 & 11 & 5 & 3 & 7\\ \frac{1}{11} & \frac{1}{10} & 1 & 4 & \frac{1}{5} & \frac{1}{7} & \frac{1}{3}\\ \frac{1}{13} & \frac{1}{11} & \frac{1}{4} & 1 & \frac{1}{7} & \frac{1}{9} & \frac{1}{5}\\ \frac{1}{7} & \frac{1}{5} & 5 & 7 & 1 & \frac{1}{4} & 3\\ \frac{1}{5} & \frac{1}{3} & 7 & 9 & 4 & 1 & 5\\ \frac{1}{9} & \frac{1}{7} & 3 & 5 & \frac{1}{3} & \frac{1}{5} & 1 \end{array}\right],\\ {\text{CR}}_{3}=0.0908<0.1,\\ B_{4}=\left[\begin{array}{ccccccc} 1 & 7 & 5 & 11 & 4 & 9 & \frac{1}{4}\\ \frac{1}{7} & 1 & \frac{1}{3} & 5 & \frac{1}{5} & 3 & \frac{1}{9}\\ \frac{1}{5} & 3 & 1 & 8 & \frac{1}{3} & 5 & \frac{1}{7}\\ \frac{1}{11} & \frac{1}{5} & \frac{1}{8} & 1 & \frac{1}{9} & \frac{1}{3} & \frac{1}{13}\\ \frac{1}{4} & 5 & 3 & 9 & 1 & 7 & \frac{1}{5}\\ \frac{1}{9} & \frac{1}{3} & \frac{1}{5} & 3 & \frac{1}{7} & 1 & \frac{1}{12}\\ 4 & 9 & 7 & 13 & 5 & 12 & 1 \end{array}\right],\\ {\text{CR}}_{4}=0.0919<0.1, \end{array}$$

where **B**_1_ is the comparison matrix of tunnel entrance collapse, **B**_2_ is the comparison matrix of slope instability, **B**_3_ is the comparison matrix of large deformation, and **B**_4_ is the comparison matrix of water inrush.

Weight vectors of the risk factor are calculated using the eigenvalue method which are as follows:

(10) $$\begin{array}{c} W_{B}=\left[0.5962\;0.2616\;0.0989\;0.0434\right],\\ W_{C}=\left[\begin{array}{c} W_{1}\\ W_{2}\\ W_{3}\\ W_{4} \end{array}\right]=\left[\begin{array}{ccccccc} 0.2569 & 0.1450 & 0.4337 & 0.0148 & 0.0250 & 0.444 & 0.0802\\ 0.1467 & 0.0143 & 0.0423 & 0.0240 & 0.0753 & 0.4354 & 0.2619\\ 0.4307 & 0.2591 & 0.0255 & 0.0141 & 0.0764 & 0.1500 & 0.0441\\ 0.2522 & 0.0435 & 0.0802 & 0.0143 & 0.1365 & 0.0243 & 0.4490 \end{array}\right], \end{array}$$

where **W**_*B*_ is the weight of the four risk events and **W**_*C*_ is the weight of each risk factor relating to different risk events.

  Step 2: identify the membership degree matrix

The membership degree matrix **R** has been calculated in Section 2.3.1

  Step 3: single risk factor fuzzy evaluation

Combining the weight of single risk factors with **R**, the single risk factor fuzzy comprehensive evaluation matrix is calculated as follows:

(11) $$\begin{array}{c} R^{\prime }=W_{C}\cdot R=\left[\begin{array}{ccccc} 0.4409 & 0.1952 & 0.1583 & 0.1541 & 0.0514\\ 0.5055 & 0.2288 & 0.2655 & 0 & 0\\ 0.0443 & 0.2085 & 0.5747 & 0.1722 & 0\\ 0.4639 & 0.5360 & 0 & 0 & 0 \end{array}\right], \end{array}$$

where **R**′ is the comprehensive judgement result combined with the risk factor weight **W**_*C*_ and the membership matrix **R**, which represents the first comprehensive evaluation.

  Step 4: multifactor fuzzy comprehensive evaluation

Taking **R**′ as a membership matrix of multifactor fuzzy comprehensive evaluation, the comprehensive evaluation value was calculated by combining with **W**_*B*_ and **R**′. The results of fuzzy comprehensive evaluation are as follows:

(12) $$\begin{array}{c} B_{Y}=W_{B}\times R^{\prime }=\left[0.4196\;0.2201\;0.2206\;0.1089\;0.0306\right], \end{array}$$

where **B**_*Y*_ is a judgement matrix of the risk event obtained by two fuzzy comprehensive evaluation processes.

This research chooses the maximum membership degree principle to identify the risk level because it is generally applicable in the course of engineering risk assessment. The risk level of the construction stage in highway Tunnel 1 portal is most likely to be level I.

  Step 5: risk probability score

Each risk evaluation level is represented by a digit, which is converted to a corresponding probability score according to Table 3, as the output value of the proposed model.

As a result, the risk score of Tunnel 1 is 10000.

Similarly, the probability of construction risk of the other 44 tunnel portals and the risk score of overall 45 tunnels can be obtained as shown in Table 4.

### 2.4. BP Neural Network Algorithm Flow

The BP neural network is a kind of a network with a teacher. The teachers are actually a training sample for training the network. The input and output mappings of the network are obtained from training samples. The sample input and the target output required by the network must be known, and the weight coefficient between each layer is determined by the input after the exact output value can be derived. In the BP neural network, the data are propagated backwards from the input layer through the hidden layer. If the output layer does not have the desired output value, the connection weight of the network is corrected from the output layer in the direction of reducing the error. The error will gradually decrease with the continuous learning of the network until the error is no longer down, and then the network training is completed.

#### 2.4.1. Determination of Learning Rate

With different learning rates, there is great influence on the performance of the established BP neural network model. The smaller the learning rate is, the slower the convergence rate is. If the learning rate is too large, the training is prone to oscillation. At present, we can only roughly determine the learning rate through experience for different issues, and the selection range is generally in the range [0.01, 0.8].

#### 2.4.2. Determination of the Number of Hidden Neurons

The performance of the BP neural network is also related to the number of hidden neurons. In general, the bigger the number of hidden neurons is, the better the network performance is. However, if the number of hidden neurons is too much, the training time may be too long. Currently, there is no ideal analytical formula for determining the number of hidden neurons. Generally, the following empirical formula is used to obtain the estimated value:

(13) $$\begin{array}{c} M=\sqrt{n+m}+a, \end{array}$$

where *M* is the number of hidden neurons, *n* is the number of input layer neurons, *m* is the number of output layer neurons, and a is a constant in the range [0, 10].

In the three-layer BP neural network, assuming that the number of input neurons is *I*, the number of hidden neurons is *J*, and the number of neurons in the output layer is *N*, the *i* neurons of the input layer are denoted as *x*_*i*_, the *j* neurons of the hidden layer are denoted as *k*_*j*_, and the *n* neurons of the output layer are denoted as *y*_*n*_. The algorithm is as follows:

Step 1: determine the structure of the network and then initialize all of the network weights and the threshold neurons in the hidden layer and output layer.
Step 2: the fuzzy sample dataset {*x*_*pl*_} and the corresponding expected output set {*y*_*pl*_} are input to the network for training, where *p* is the number of samples and *l* is the number of input vectors.
Step 3: calculate the output *O* of each layer. The input and output are the same in the input layer, that is, *O*_*pi*_=*x*_*pi*_, where *x*_*pi*_ is the *i* value of the *p* sample; the output operation of the neuron is *O*_*pi*_=*f*(∑*w*_*pi*_ − *θ*_*i*_) for the hidden layer and the output layer, where *O*_*pi*_ is the output of neuron *i* and the input of neuron *j*; the neurons in the hidden layer use the sigmoid function, that is,
  (14) $$\begin{array}{c} f\left(x\right)=\frac{1}{\left(1+e^{-x}\right)}. \end{array}$$
Step 4: calculate the value of each layer. The error of the output layer is expressed as *δ*_*pj*_=(*y*_*pj*_ − *O*_*pj*_)*O*_*pj*_(1 − *O*_*pj*_); the error of the hidden layer is expressed as *δ*_*pj*_=*O*_*pj*_(1 − *O*_*pj*_)∑*δ*_*pj*_ *w*′_*pj*_.
Step 5: adjust the weight value by using *w*′_*ji*_(*t*+1)=*w*′_*ji*_(*t*)+*αδ*_*pj*_ *O*_*pj*_, where *α* is the learning speed.
Step 6: calculate the error by using *E*_r_=12∑*p*∑*k*(*O*_*pk*_ − *y*_*pk*_)^2^. If *E* is less than the expected error value, the network training will end. Otherwise, the value will return to Step 3 and then continue training until the error value satisfies the preset value.

**Figure 3:**
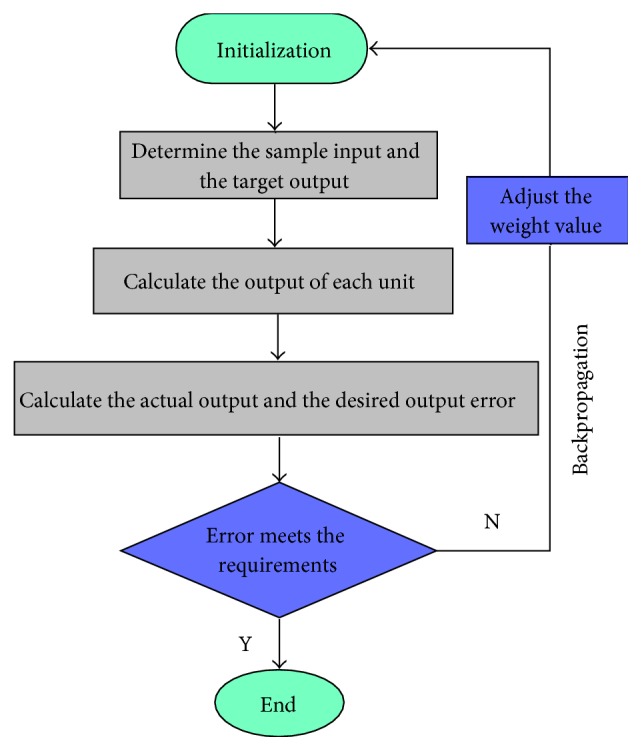
The flow diagram of the BP algorithm.

The flow diagram of the BP algorithm is shown in Figure 3.

### 2.5. BP Fuzzy Neutral Network Model Training

Forty-five samples data from the 50 tunnel samples data are selected, which have been obtained in Section 2.3, of highway tunnel portal construction for network training. The remaining five samples data were compared with the results which are obtained from the model as test samples. The test results are shown in Table 5.

By comparing the test results with the expected results, the difference between the two values is taken as the error, and the error of each object is obtained. The cumulative error of the five subjects is 2.06%, and the prediction accuracy of the network model is 97.94%, which indicates that the BP fuzzy neural network model has a high prediction accuracy.

## 3. Case Study

### 3.1. Hydrogeological Condition

The Tiefodian highway tunnel is connected to Baoji and Hanzhong in Shaanxi Province of China located in the south of Tiefodian Town, the west of the National Highway 316. The tunnel is composed of two separate tunnels, which belong to short tunnels. The left tunnel being 135 m long has a mileage pile number of ZK164 + 570–ZK164 + 705. The right tunnel is 185 m long and has a mileage pile number of YK164 + 550–YK164 + 735.

The tunnel is perpendicular to the slope surface with no bias, and the portal of this tunnel lies in the valley foot on the right bank of Bao River. The depth of the tunnel portal is lower than 36.0 m, which belongs to the shallow tunnel. The tunnel span is 16.0 m. The inclination angle of the stable natural slope is 41°. The lithology along the tunnel is completely weathered gneiss. During the construction of tunnel portal excavation, the collapse and the slope instability are easily caused. Therefore, there is a certain degree of difficulty in excavating the tunnel portal.

**Figure 4:**
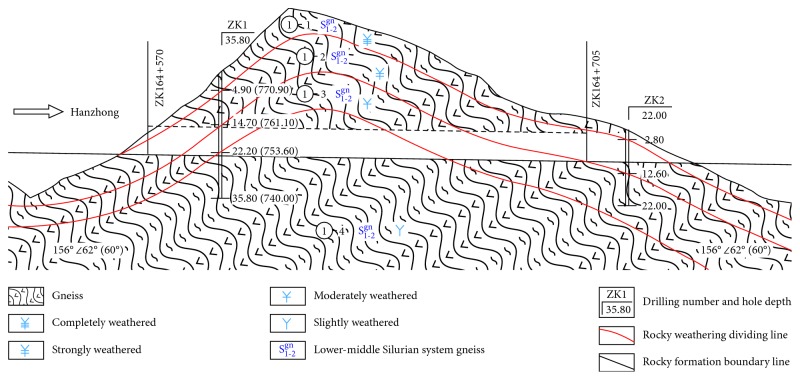
Longitudinal profile of the left tunnel.

The distance between the left tunnel exit and right tunnel exit is 30 m. The surrounding rock type is completely weathered gneiss. The surrounding rock of the right tunnel portal and left tunnel portal is both of level V. Take the portal section of the left tunnel as the research object for risk assessment. The longitudinal profile of the left tunnel is shown in Figure 4.

### 3.2. Construction Method of the Tunnel Portal

**Figure 5:**
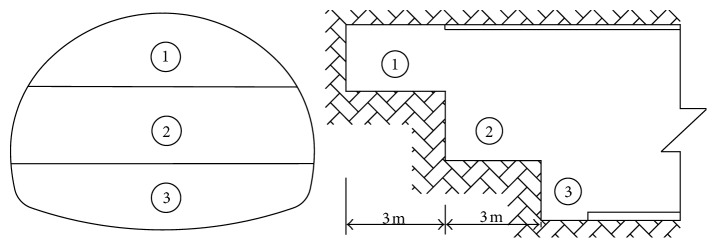
Construction steps of the bench method.

Tunnel portals are constructed with the bench method, which is shown in Figure 5. The tunnel excavation *C* is listed as follows:

  Step 1: in the arch of the tunnel, the advanced small pipe with grouting reinforcement strata is used.
  Step 2: the upper bench is excavated.
  Step 3: the initial support of the bolt, steel frame, and shotcrete is constructed at the upper bench.
  Step 4: the lower bench is excavated.
  Step 5: the initial support of the bolt, steel frame, and shotcrete is constructed at the lower bench.
  Step 6: the invert arch is excavated.
  Step 7: the concrete of the invert arch is constructed. The excavation distance of each part is 3 m.

### 3.3. Risk Evaluation Index

The construction risk of the Tiefodian tunnel portal is evaluated according to the proposed model in Section 2. The sample data are employed by the abovementioned fuzzy method in Section 2.2, as shown in Table 6.

Taking those input data into the evaluation model which has been trained completely in Section 2, the expected output results are as follows:

(15) $$\begin{array}{c} Y=\left[0.0000\;0.0000\;0.0084\;0.9403\;0.0513\right]. \end{array}$$

Obviously, the risk probability level of the Tiefodian tunnel is level IV, which belongs to high risk.

### 3.4. Comparison and Discussion

In order to verify the accuracy of the proposed model, FAHP is used to calculate the risk probability level of Tiefodian tunnel portal construction. According to the FAHP method described in Section 2.2, the total ranking results of the Tiefodian tunnel are shown in Table 7.

The risk value with fuzzy comprehensive evaluation is as follows:

(16) $$\begin{array}{c} B=\left[0.0126\;0.2332\;0.2608\;0.3798\;0.1134\right]. \end{array}$$

The result shows that the risk level is level IV, which belongs to high risk.

According to Table 6, tunnel entrance collapse (*B*_1_) is most likely to happen due to the risk of Tiefodian tunnel portal construction. The largest relative weight coefficient is 0.2756, which indicates the construction method. Similarly, the smallest relative weight coefficient indicates unsymmetrical load. In accordance with the results, the risk factors in the Tiefodian tunnel portal section are ordered according to importance as follows: surrounding rock level, support parameters, rainfall and groundwater, cross-sectional size, buried depth, and unsymmetrical load.

The BP fuzzy neural network method and FAHP method are used to calculate the risk probability level in Tiefodian tunnel portal construction. The results are shown in Table 8.

The BP fuzzy neural network method and FAHP method are the same as the evaluation results of the risk probability grade in the construction stage of the Tiefodian tunnel. The advantages of the BP fuzzy neural network method are as follows: the BP fuzzy neural network method has the characteristics of strong fault tolerance and self-adaptability, which can overcome the inaccuracy of the result quantification of each risk factor in FAHP. Coupled with the fuzzy algorithm, the BP fuzzy neural network method is applied to form a composite evaluation system, which makes the results more reasonable and closer to the actual situation.

### 3.5. Risk Control Measures

**Figure 6:**
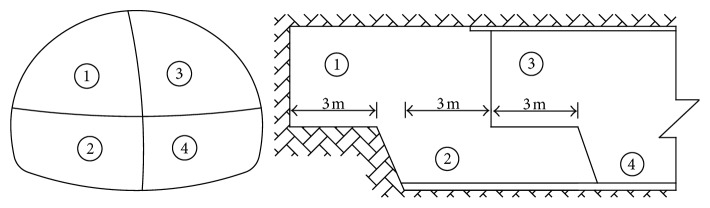
Construction of the left tunnel portal by the CD method.

The risk probability level of the construction of Tiefodian tunnel portals is grade IV, which indicates high risk. Risk control measures should be applied to the construction to ensure the stability of the tunnel. In view of the risk assessment results in Section 3.4, the risk control measures are proposed as follows:

1. Adjust the construction method: the original construction method is the bench method, which can cause the surrounding rock disturbance. Therefore, we exchange the bench method for the centre diaphragm (CD) method. The excavation footage is no greater than 3.0 m, and the distance between different excavation parts is at 9.0 m to maintain tunnel stability. Meanwhile, the anchorage, primary lining, and invert arch are constructed in the tunnel. When the length of the invert arch is 12 m, the secondary lining is installed. The blasting charge should be strictly controlled in the portal section to reduce the blasting excavation risk. Finally, the dynamic monitoring of tunnel deformation should be strengthened during the construction process. The construction of the CD method is shown in Figure 6.
2. Reinforcement of surrounding rock: ensuring the stability of the tunnel portal is difficult because of the poor quality of the rock mass, the thinner cover depth, and rainfall in the Tiefodian highway tunnel. As a result of this, the presupport measures should be improved before the excavation of the tunnel. Actually, the pipe roof has a good effect as a presupport measure in the construction of the tunnel portals. A 30 m long pipe roof, which consists of a total of 44 seamless steel pipes (Φ108 × 6 mm) with a construction scope of 2 × 57°, is suggested as a presupport measure of the tunnel portal on the basis of the abovementioned analysis. The length of single pipe sections is 3 or 6 m. They are connected at the joints by 150 mm long threaded sections. With the use of the umbrella arch as a guide wall, the pipe roof is constructed along the outer contour line of the open cut.
3. Strengthening the supporting parameters: the supporting parameters are important in avoiding tunnel face instability and great deformation of the tunnel portals. Therefore, the diameter of the rock bolt is improved to Φ28, and the length is increased to 4.5 m. The I22b steel frame in H shape is adopted, and the length of the pregrouting bolt is added to 5.0 m.
4. In the construction process, the monitoring work should also be carried out simultaneously, and the measured data should be backanalyzed. The stability of the surrounding rock is determined by the analysis results of monitoring data. And the support parameters are adjusted to ensure the safety of construction.

## 4. Model Validations

The deformation of the surrounding rock and the force of the supporting structure were simulated based on the construction conditions of the Tiefodian tunnel. The rationality of the proposed model and the risk measures was proved by simulating and analyzing with the MIDAS/GTS finite-element analysis software. The ZK164 + 570 section and ZK164 + 600 section are selected as the object of numerical simulation which belong to the Tiefodian tunnel portal.

According to the Saint-Venant principle, tunnel excavation has little effect on the surrounding rock which is located in 3–5 times the diameter. The model size is taken as *X* × *Y* × *Z* = 100 m × 30 m × 90 m in this simulation. The main design parameters are selected as shown in Table 9.

### 4.1. Numerical Simulations before Risk Control Measures

**Figure 7:**
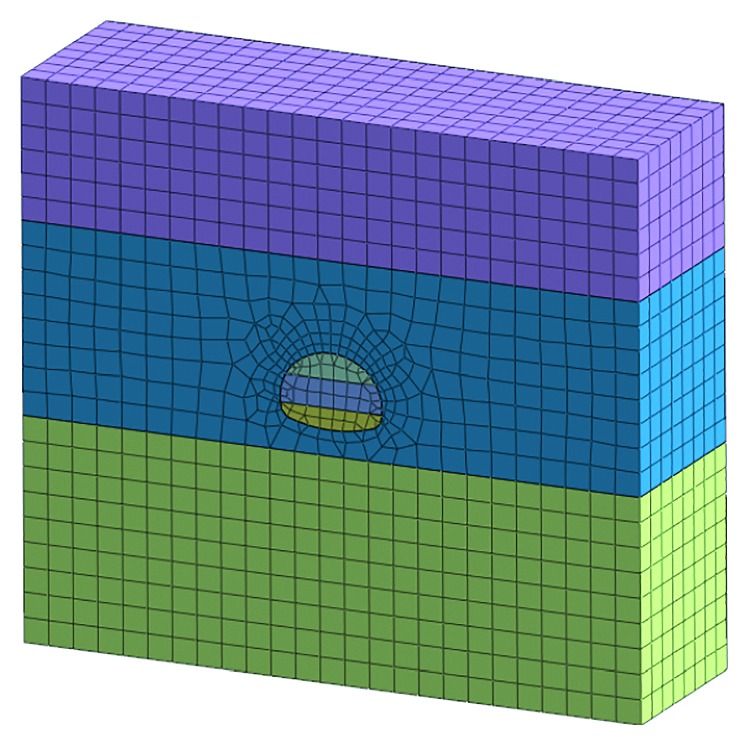
Model meshing before risk controls.

**Figure 8:**
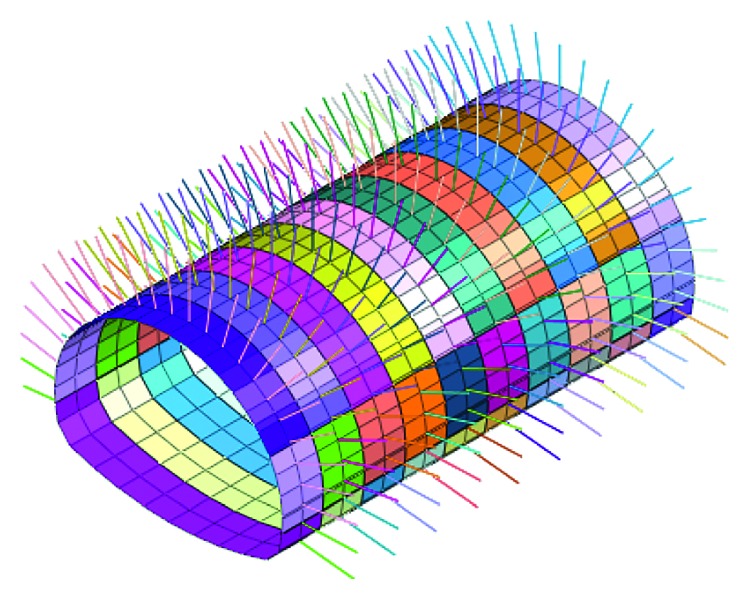
Spray anchor support before risk controls.

This simulation is based on the bench method before the risk control measures in the construction. The model meshing and the supporting structure simulation are established as shown in Figures 7 and 8.

**Figure 9:**
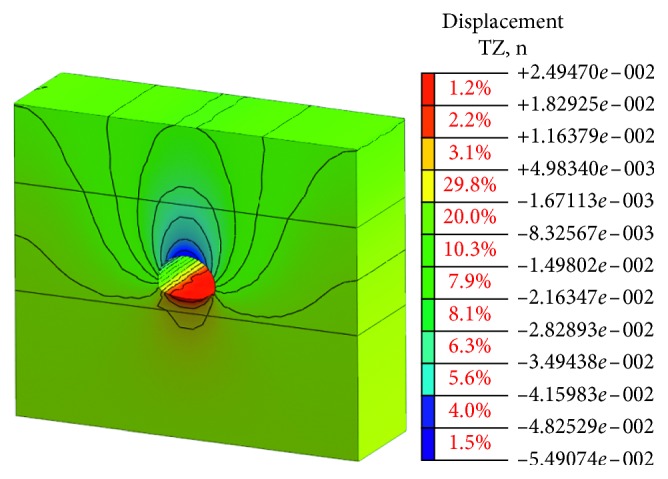
Vault settlement value.

**Figure 10:**
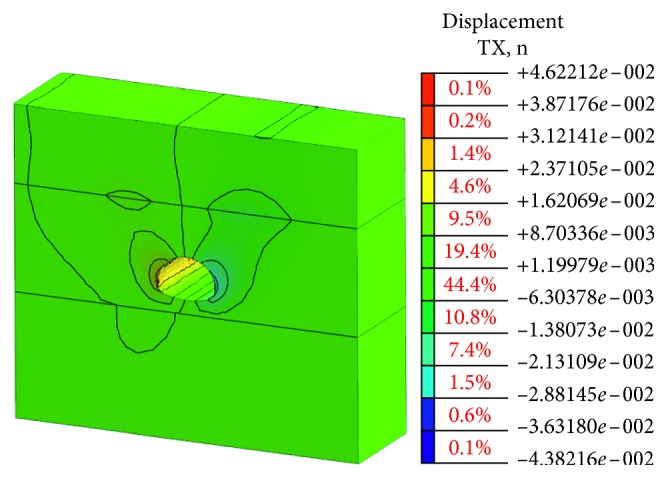
Horizontal convergence value.

The stress state of the surrounding rock is redistributed and deformed toward the temporary surface with the excavation process of the tunnel cavern. The deformation of the cavern can be the most intuitive and convenient response to the deformation state of the surrounding rock. The value of vault settlement and horizontal convergence is a typical observation project to reflect the deformation of cavern. The final deformation of the tunnel portal with the bench method before the risk control is shown in Figures 9 and 10.

Obviously, the deformation of the surrounding rock after tunnel excavation is symmetrical, which is mainly reflected in the vault, and the sidewall converges inward. The maximum vault settlement value is about 54.91 mm, and the maximum arched part has a certain amount of spring back about 24.95 mm due to the unloading effect. The horizontal convergence value of the surrounding rock at the left and right sidewalls is about 46.22 mm. The vault settlement value and the horizontal convergence value of surrounding rock are larger than the specification requirement [23]. There is a risk in the tunnel portal construction, and the corresponding risk control measures should be taken to strengthen the tunnel and ensure the safety of the tunnel.

### 4.2. Numerical Simulations after Risk Control Measures

**Figure 11:**
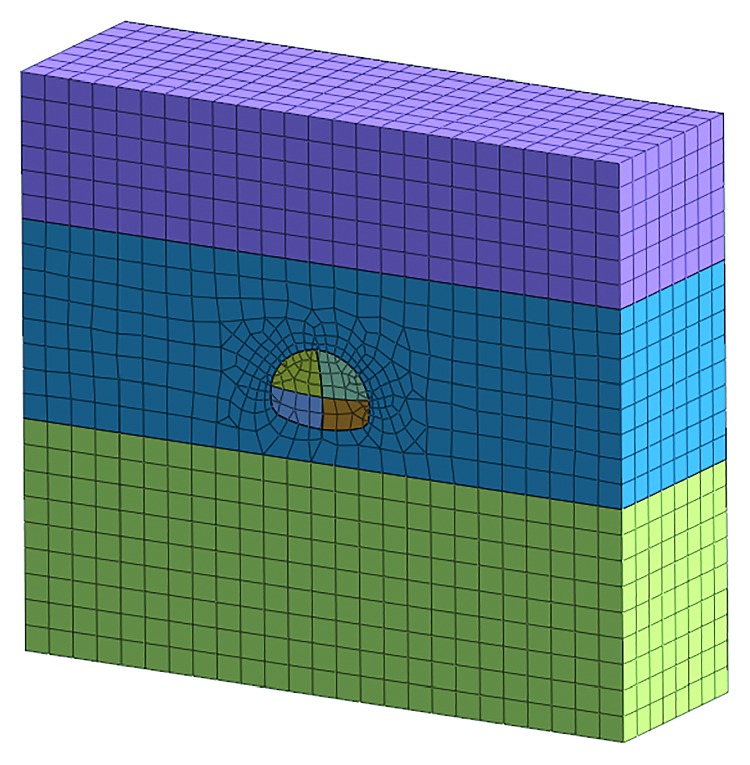
Model meshing after risk controls.

**Figure 12:**
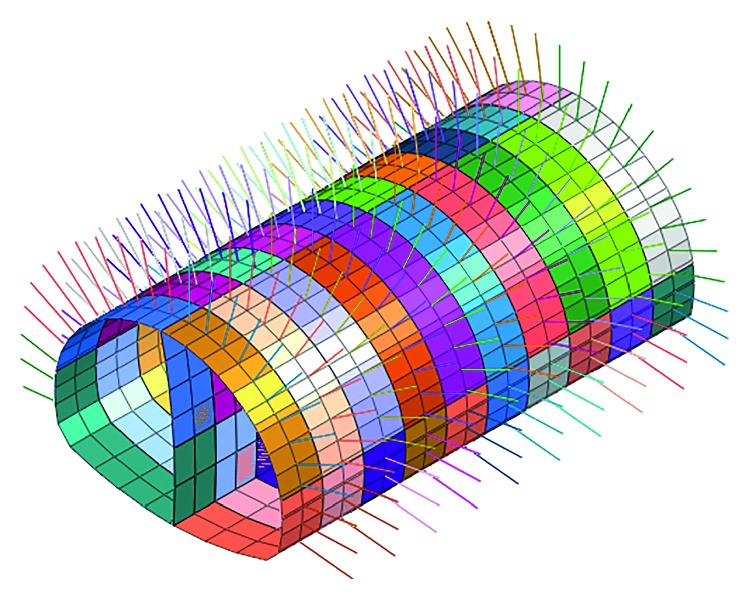
Spray anchor support after risk controls.

Adjust the construction method and support parameters according to the importance of risk factors; this simulation is based on the CD method after the risk control measures in the construction. The model meshing and the support structure simulation are established as shown in Figures 11 and 12.

**Figure 13:**
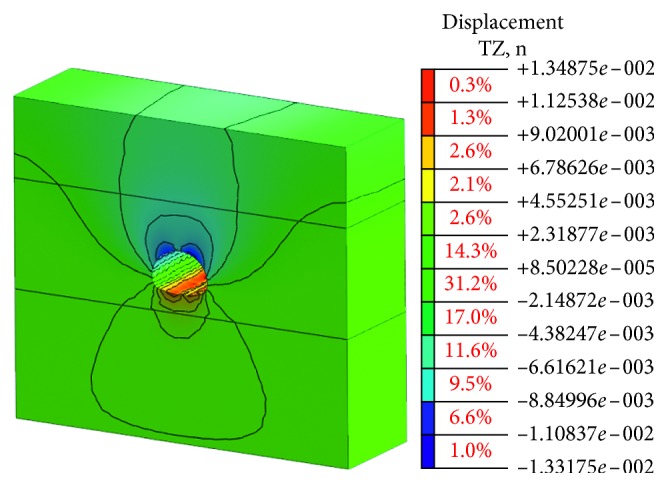
Vault settlement value.

**Figure 14:**
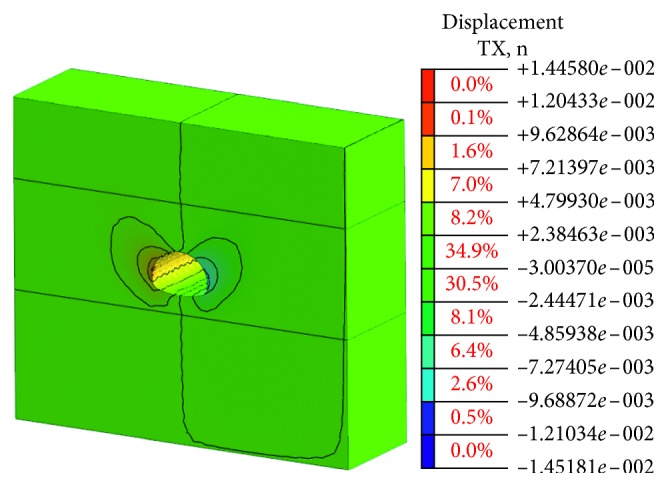
Horizontal convergence value.

Through the numerical simulation analysis, the vault settlement and the horizontal convergence cloud diagram of surrounding rock at each construction stage obtained are shown in Figures 13 and 14.

It can be seen from Figures 3 and 4 that the deformation value of surrounding rock is declining after adopting the CD construction method and optimizing the support parameters. The maximum value of the vault settlement is about 13.32 mm. The horizontal convergence value of the surrounding rock at the left and right sidewalls is about 13.49 mm.

### 4.3. Discussion

The numerical simulations of two cases are listed and analyzed in order to verify the effectiveness of the proposed measures. The details are shown in Table 10.

It can be seen from Table 10 that the values of the vault settlement before and after the risk control are 54.91 mm and 6.27 mm, respectively. The values of vault settlement after risk controls are far less than those before risk controls. Similarly, the values of horizontal convergence after risk controls are also far less than those before risk controls. Therefore, the comparison results indicate that the risk evaluation model established in this paper is accurate to evaluate the construction risk of the tunnel portal and the proposed risk control measures are feasible (Table 11).

## 5. Conclusion

This paper develops a BP fuzzy neural network model based on fuzzy theory and BP neural network to handle the vagueness and subjectivity in risk evaluation of highway tunnel portal construction. A risk evaluation model which is obtained from historical data of 50 tunnels is established by combining the fuzzy method with the BP neural network. The proposed model is applied for the risk assessment of the Tiefodian tunnel. The results show that the risk evaluation level is IV and slope instability is the greatest impact index among four risk events. Based on above analysis, we conclude that supporting parameters, rainfall and groundwater, and surrounding rock level precede the others. At the same time, this model is confirmed to be available in risk evaluation of the tunnel portal by using the fuzzy analytic hierarchy process (FAHP). According to the evaluation results, corresponding risk control measures are suggested and taken. Besides, numerical simulation is carried out before and after the implementation of risk measures, respectively. The rationality of the proposed risk evaluation model is proved by comparing the numerical simulation results.

However, the proposed method is still a semiquantitative risk evaluation method. Because of the complicated geological conditions and uncertainties of tunnel construction, the risk identification is based on the rich experience of experts in this paper. Although the main risk factors can be identified, some less influence of the risk factors has the probability be missed. Moreover, the number of tunnel samples for the proposed model needs to be further expanded to improve the accuracy of risk evaluation.

## Figures and Tables

**Table 1:** The input data of Tunnel 1.

*X*_1_	*X*_2_	*X*_3_	*X*_4_	*X*_5_	*X*_6_	*X*_7_	*X*_8_	*X*_9_	*X*_10_	*X*_11_	*X*_12_	*X*_13_	*X*_14_	*X*_15_	*X*_16_	*X*_17_	*X*_18_	*X*_19_	*X*_20_	*X*_21_	*X*_22_	*X*_23_	*X*_24_	*X*_25_	*X*_26_	*X*_27_	*X*_28_
0.136	0.200	0.344	0.360	0.328	0.256	0.344	0.360	0.256	0.280	0.352	0.360	0.232	0.360	0.168	0.224	0.336	0.280	0.280	0.208	0.328	0.360	0.360	0.360	0.328	0.296	0.304	0.288

**Table 2:** The average random consistency indicator.

Matrix order	3	4	5	6	7	8	9
RI	0.58	0.90	1.12	1.24	1.32	1.41	1.46

**Table 3:** The correspondence between the risk level and score.

Risk level	I	II	III	IV	V
Score	10000	01000	00100	00010	00001

**Table 4:** The risk probability score of sample tunnels.

Tunnel	1	2	3	4	5	6	7	8	9
Score	10000	00100	00100	01000	00100	00010	01000	01000	00100

Tunnel	10	11	12	13	14	15	16	17	18
Score	01000	00010	00100	00010	01000	00100	00100	01000	00100

Tunnel	19	20	21	22	23	24	25	26	27
Score	00100	00100	00010	00100	00010	00100	00010	00100	00100

Tunnel	28	29	30	31	32	33	34	35	36
Score	00100	00100	01000	00010	01000	01000	00010	00010	00100

Tunnel	37	38	39	40	41	42	43	44	45
Score	00100	01000	00010	00010	00100	00100	10000	00100	00010

**Table 5:** Comparison of network test results.

Tunnel	Test results of the fuzzy neural network					Model result	Cumulative error	Realistic risk level
	Output 1	Output 2	Output 3	Output 4	Output 5			
46	0.0000	0.0001	1.0000	0.0000	0.0000	0 0 1 0 0	0.01%	III
47	0.0000	0.0032	0.9992	0.0000	0.0000	0 0 1 0 0	0.40%	III
48	0.0000	0.0001	0.9497	0.0016	0.0000	0 0 1 0 0	5.20%	III
49	0.0000	0.0003	0.9854	0.0001	0.0000	0 0 1 0 0	1.50%	III
50	0.0000	0.9847	0.0149	0.0001	0.0015	0 1 0 0 0	3.18%	II

**Table 6:** The input data of the Tiefodian tunnel.

*X*_1_	*X*_2_	*X*_3_	*X*_4_	*X*_5_	*X*_6_	*X*_7_	*X*_8_	*X*_9_	*X*_10_	*X*_11_	*X*_12_	*X*_13_	*X*_14_	*X*_15_	*X*_16_	*X*_17_	*X*_18_	*X*_19_	*X*_20_	*X*_21_	*X*_22_	*X*_23_	*X*_24_	*X*_25_	*X*_26_	*X*_27_	*X*_28_
0.224	0.280	0.088	0.296	0.168	0.096	0.152	0.232	0.360	0.232	0.304	0.168	0.144	0.152	0.248	0.280	0.136	0.160	0.336	0.144	0.136	0.160	0.280	0.152	0.256	0.136	0.176	0.184

**Table 7:** The total ranking of risk factors.

Risk factors	*B*_1_	*B*_2_	*B*_3_	*B*_4_	Total ranking
	0.5962	0.2616	0.0989	0.0434	
*C*_1_	0.2569	0.1467	0.4307	0.2522	0.2451
*C*_2_	0.1450	0.0143	0.2591	0.0435	0.1177
*C*_3_	0.4337	0.0423	0.0255	0.0802	0.2756
*C*_4_	0.0148	0.0240	0.0141	0.0143	0.0171
*C*_5_	0.0250	0.0753	0.0764	0.1365	0.0481
*C*_6_	0.0444	0.4354	0.1500	0.0243	0.1563
*C*_7_	0.0802	0.2619	0.0441	0.4490	0.1402

**Table 8:** Comparison of evaluation methods.

Evaluation target	BP fuzzy neural network method	FAHP method
Risk result	(0.0000, 0.0000, 0.0084, 0.9403, 0.0513)	(0.0126, 0.2332, 0.2608, 0.3798, 0.1134)
Risk level	IV (high risk)	IV (high risk)

**Table 9:** The model parameters after optimization.

Formation or structure	Elastic modulus *E* (GPa)	Poisson's ratio (*μ*)	Bulk density (kN/m^3^)	Cohesion (kPa)	Internal friction angle (°)
Fully weathered gneiss	0.2	0.4	18	50	28
Strong weathering gneiss	0.5	0.35	20	200	32
Weathering gneiss	1	0.3	22	320	35

**Table 10:** The comparison of the deformation value.

Deformation value	Evaluation index		
	Vault settlement (mm)	Horizontal convergence (mm)	Specification allowed (mm)
Before the risk control	54.91	46.22	40
After the risk control	6.27	13.32	40

**Table 11:** The historical data of the 50 tunnels.

Number	Tunnel name	Number of one-way lanes	Location	Surrounding rock level	Tunnel length (m)	Maximum depth (m)
1	Aofeng Mountain Tunnel	Three lanes	Fuzhou	IV	1867	—
2	Lake Yun No. 1 Tunnel	Two lanes	Mianzhu	V	3555	400
3	Mountain Hu Tunnel	Two lanes	Nanjing	IV	1645	466
4	Cang Ridge Tunnel	Two lanes	Taijin	IV	7530	768
5	Jiuling Mountain Tunnel	Two lanes	Wuji	V	5473	887
6	Qingshan Hillock Tunnel	Two lanes	Changsha	V	1245	80
7	Xuefeng Mountain Tunnel	Two lanes	Anhua	IV	7039	780
8	Anyuan Tunnel	Two lanes	Anyuan	V	6868	470
9	Lujialing Tunnel	Two lanes	Chongqing	IV	6664	701
10	Wujian Ridge Tunnel	Two lanes	Yongzhang	V	1050	197
11	Mayazi Tunnel	Two lanes	Wuguan	V	9007	714
12	Shanziding Tunnel	Two lanes	Meida	V	500	110
13	Luwan Tunnel	Three lanes	Lishui	IV	708	170
14	Mountain Mao Tunnel	Three lanes	Changning	V	1628	50
15	Tongzhou Tunnel	Two lanes	Yongjia	IV	493	102
16	Wuzhi Mountain Tunnel	Three lanes	Leshan	V	3923	800
17	Xiang River Tunnel	Two lanes	Huangyuan	IV	1858	300
18	Zhongtiao Mountain Tunnel	Two lanes	Jiezhou	V	7423	605
19	South Village Tunnel	Two lanes	Nancun	IV	6787	153
20	Wolonggang Tunnel	Three lanes	Beijing	V	420	35
21	Liujiapai Tunnel	Two lanes	Liujiapai	V	1233	136
22	Nan Yanmenguan Tunnel	Two lanes	Shanyin	IV	5247	600
23	Foling Tunnel	Two lanes	Linfen	IV	8803	762
24	Xilingjing Tunnel	Two lanes	Taijia	V	6555	711
25	Yanmenguan Tunnel	Two lanes	Xizhou	V	5183	600
26	Yangtou Mountain Tunnel	Two lanes	Qianjiang	V	5385	466
27	Sumu Mountain Tunnel	Three lanes	Huhetaote	IV	3213	—
28	Xuefeng Moutain Tunnel	Two lanes	Shaoyang	V	6958	840
29	Taining Tunnel	Two lanes	Taining	IV	7039	485
30	Leigong Mountain Tunnel	Three lanes	Xiamen	V	3433	253
31	Zhengjiayuan Tunnel	Two lanes	Zhashui	V	2037	189
32	Foyangling Tunnel	Four lanes	Binzhou	IV	3904	70
33	Baiyangwan Tunnel	Three lanes	Hangzhou	V	1400	52
34	Wanxichong Tunnel	Three lanes	Kunming	V	7980	754
35	Mountain Tiger Tunnel	Four lanes	Jinan	V	1880	276
36	Jianping Tunnel	Two lanes	Tongchuan	IV	1287	240
37	Yanling Mountain Tunnel	Two lanes	Hangzhou	IV	1250	178
38	Jinzhuwan Tunnel	Three lanes	Chongqing	V	1322	255
39	Yangzong Tunnel	Three lanes	Yuxi	V	2727	141
40	Magongci Tunnel	Four lanes	Zibo	IV	655	86
41	Wulidun Tunnel	Two lanes	Rucheng	V	2380	980
42	Zhenbao Tunnel	Two lanes	Boshan	IV	2880	32
43	Daiyuling No. 2 Tunnel	Two lanes	Zhuanghe	V	2930	262
44	Shimenya Tunnel	Two lanes	Yichang	V	7524	894
45	Queer Mountain Tunnel	Two lanes	Ganzi	IV	7079	700
46	Xueshanliang Tunnel	Two lanes	Abazhou	IV	6950	598
47	Ziyang Tunnel	Two lanes	Ziyang	V	7938	904
48	Baihua Mountain Tunnel	Three lanes	Wuding	V	1620	—
49	Jiaodongao Tunnel	Two lanes	Ningbo	IV	2185	410
50	Shigu Tunnel	Three lanes	Dongguan	IV	4011	500
